# Expression of Functional Recombinant Human Tissue Transglutaminase (TG2) Using the Bac-to-Bac Baculovirus Expression System

**DOI:** 10.15171/apb.2016.08

**Published:** 2016-03-17

**Authors:** Yaghoub Yazdani, Shahram Azari, Hamid Reza Kalhor

**Affiliations:** ^1^ Infectious Diseases Research Center and Laboratory Sciences Research Center, Golestan University of Medical Sciences, Gorgan, Iran.; ^2^ Department of Molecular Medicine, Faculty of Advanced Medical Science Technologies, Golestan University of Medical Sciences, Gorgan, Iran.; ^3^ Biochemistry Research Laboratory, Department of Chemistry, Sharif University of Technology, Tehran, Iran.

**Keywords:** Tissue transglutaminase (TG2), Baculovirus, Bac-to-Bac, Sf9, Protein expression

## Abstract

***Purpose:*** Tissue transglutaminase (TG2) is a unique multifunctional enzyme. The enzyme possesses enzymatic activities such as transamidation/crosslinking and non-enzymatic functions such as cell migration and signal transduction. TG2 has been shown to be involved in molecular mechanisms of cancers and several neurodegenerative diseases such as Alzheimer’s disease. The present study aimed at cloning and expression of full length human TG2 in Bac-to-Bac baculovirus expression system and evaluation of its activity.

***Methods:*** pFastBac HTA donor vector containing coding sequence of human TG2 was constructed. The construct was transformed to DH10Bac for generating recombinant bacmid. The verified bacmid was transfected to insect cell line (Sf9). Expression of recombinant TG2 was examined by RT-PCR, SDS-PAGE and western blot analysis. Functional analysis was evaluated by fluorometric assay and gel electrophoresis.

***Results:*** Recombinant bacmid was verified by amplification of a band near to 4500 bp. Expression analysis showed that the enzyme was expressed as a protein with a molecular weight near 80 kDa. Western blot confirmed the presence of TG2 and the activity assays including flurometric assay indicated that the recombinant TG2 was functional. The electrophoresis assay conformed that the expressed TG2 was the indeed capable of crosslinking in the presence of physiological concentration calcium ions.

***Conclusion:*** Human TG2 was expressed efficiently in the active biological form in the Bac-to-Bac baculovirus expression system. The expressed enzyme could be used for medical diagnostic, or studies which aim at finding novel inhibitors of the enzymes . To best of our knowledge, this is probably the first report of expression of full length human tissue transglutaminase (TG2) using the Bac-to-Bac expression system.

## Introduction

Transglutaminases (TGs) are a protein family consisting of 9 members in mammals: factor XIII, protein band 4.2, and transglutaminases 1 to 7.^1-3^ They belong to a papain-like superfamily.^1,4^ One of the most attractive members of this family is transglutaminase 2.

Transglutaminase 2 (TG2) also known as tissue transglutaminase (tTG) is a monomeric protein with 76-80 kDa molecular mass which ubiquitously is expressed in many tissues. The enzyme has been found in the cytosol and other cellular compartments such as mitochondria,^5^ and extracellular matrix. TG2, as a multifunctional protein, has a number of calcium dependent enzymatic activities such as transamidation/crosslinking^6,7^ (Table 1). TG2 interacts non-covalently with several cellular and extracellular matrix (ECM) proteins such as fibronectin,^4^ integrin,^6^ and heparan sulfates.^8^ The enzyme has been involved (calcium independently) in multiple cellular processes for instance cellular proliferation and signal transduction.^5,9^ TG2 has also been implicated in some of diseases such as celiac disease,^10^ certain types of cancer,^7^ and several neurodegenerative diseases including Alzheimer’s disease (AD) (Table 1).^11,12^ TG2 has been found as a novel pharmacological target in neurodegenerative diseases.^13^ Despite of extensive studies that have been done on different aspects of TG2 specially in physiopathology of diseases,^2,7,14^ its functions, activation, and novel inhibitors for the enzyme has not been completely worked out. For this reason, we have aimed at the subcloning and expression of TG2 enzyme in this study.

Proteins produced in the baculovirus system are very similar to human proteins in terms of post-translational modifications (e.g., N- and O-glycosylation, phosphorylation) and biological activity. Moreover, the baculovirus expression vector system (BEVS) is the preferred expression system for production of large recombinant proteins.^15^ Bac-to-Bac technique is an efficient and widely used method in BEVS.^16^ In the present study, the expression of full length recombinant human tissue transglutaminase (TG2) is reported in the Bac-to-Bac baculovirus expression system, for the first time, and its activity has been also evaluated.

**Table 1:** Summary of TG2 characteristics and properties

**Protein name**	**Gene/Map locus**	**Residues/Molecular Mass (kDa)**	**Structure**	**Tissue localization**	**Chemical reaction**	**Physiological functions**	**Related disease**
Transglutaminase 2 [TG2, tissue TG, TGc, tTG]	TGM2/ 20q11-12	686-687/ 74-80	4 domain: β-sandwich catalytic core β-barrel 1 β-barrel 2	Cytosolic, nucleus, mitochondria, cell membrane	Transamidation/ crosslinking, deaminase GTPase, ATPase isomerase	Cell adhesion, migration, growth, proliferation, differentiation, cell signaling,	Coeliac disease, neurodegenerative disease [Alzheimer, Huntington, Parkinson] cancer, metastasis

## Materials and Methods

### Reagents, cells and Antibodies

All restriction enzymes, X-gal, and IPTG were obtained from Fermentas (Lithuania). dNTP and Taq DNA polymerase were purchased from Roche (Germany). Culture mediums were purchased from Gibco (USA). Cellfectin II and TRIZOL were purchased from Invitrogen (USA). Coomassie brilliant blue, HCL, NaOH and boric acid were from Merck (Germany). Nitrocellulose membrane from Schleicher & Schuell BioScience (Germany). Gentamicin, Tetracycline, Kanamycin, Tris, SDS, Protease inhibitor, Laemmli buffer, DAB, dansylcadaverine, N,N dimethyl casein, EDTA, Cacl_2_ and DTT were obtained from Sigma (USA). Primers were synthesized by Pishgam biotech (Iran). cDNA synthesis were performed by Takara kit (Japan). *Spodoptera frugiperda* (Sf9) cells were from National Cell Bank of Iran (NCBI, Pasteur Institute of Iran). Recombinant human transglutaminase 2/TGM2 was purchased from R&D systems (USA). Anti-TG2 antibody was from Abcam (UK) and anti-rabbit IgG was obtained from Santa Cruz Biotechnology (USA).

### Plasmid Construction of the recombinant tissue transglutaminase (TG2)

Human TG2 cDNA was obtained from our previous study.^17^ The TG2 cDNA that was cloned using *EcoRI*, and *XhoI* into pET-28, was digested with the same restriction enzymes and the cleaved insert containing the entire TG2 cDNA was purified from the gel and subcloned into pUC57. After confirming correct clones, the TG2/pUC57 plasmid digested with *EcoRI* and *HindIII* and the insert subcloned into pFastBacHTA vector. This vector adds a hexa-histidine tag (His-tag) to the N-terminal of the expressed protein.

Double digestion using *EcoRI*, and *HindIII* restriction enzymes and gene sequencing were used for confirmation the correct TG2 subcloning and coding sequence. The integrity of final construct was also confirmed again by PCR using specific primers 5'CCGGAATTCATGGCCGAGGAGCTGGTCTTAGAGAGG3' (TG2-forward) and 5'AACCTCGAGTTAGGCGGGGCCAATGATGACATTCC3' (TG2-reverse).^17^ For PCR, the reaction mixture (25 μL) was composed of 15 ng extracted plasmid, 1× PCR buffer, 200 μM each dNTP, 1.5 unit of Taq DNA polymerase and 0.6 μM each primers. The PCR was performed by 35 cycles of 94 °C for 30 s, 57 °C for 40 s and extension at 72 °C for 2.5 min.

### Generation of recombinant baculoviruse

The donor plasmid (pFastBac HTA) containing the TG2 coding sequence transformed into DH10 bacteria. The DH10 bacteria contain the baculovirus genome as a bacmid DNA and helper plasmid. As a result of homologous recombination, recombinant bacmid was produced.^18,19^

The transformed DH10Bac bacteria were grown for 48-72 h at 37 °C and isolated white colonies on selective LB agar plates containing 7 µg/ml gentamicin, 10 µg/ml tetracycline, 50 µg/ml kanamycin, 100 µg/ml X-gal, and 40 µg/ml IPTG. To ensure the verification of correct homologous recombination, gene amplification method using pUC/M13 forward and reverse primers was performed. The verified recombinant bacmid was used for baculovirus production.^18^

*Spodoptera frugiperda* (Sf9) insect cells that were adapted to serum-free medium, incubated at 27 °C without CO_2_ and the cells were grown in SF-900 II SFM medium lacking in antibiotics and antimycotics reagents. Transfection was performed by using the cells, 2μg verified recombinant bacmid DNA from previous step, and 10μl Cellfectin II reagent in 1 mL SF-900 II SFM without antibiotics following manufacturer's instruction as reported in previous study.^18^ After incubating at 27 °C for 5 h, the transfection mixture was changed with 2.5 ml serum-free medium. Transfected cells were allowed to grow in monolayer culture and were observed daily for signs of viral infection and cytopathic effects.

### Expression of recombinant transglutaminase

For protein expression, Sf9 cells (10^6^ cells) were transfected with recombinant baculovirus. Preliminary assessment showed that protein expression was optimal when cells were incubated for 96 h at a multiplicity of infection (MOI) of 5. After infection, cells were washed by cold PBS and lysed with cell lysate buffer (62.5 mM Tris-HCl, pH 6.8, 2% SDS) supplemented with protease inhibitor cocktail. An equal volume of Laemmli buffer (2X) was added to the sample, then samples were heated at 100 °C for 5 min and then the samples were cooled. Separation of proteins was done by SDS-PAGE (10%) for 90 min at 120 V. Then, proteins were stained with Coomassie brilliant blue R-250.

### RT-PCR 

Total cellular RNA was prepared from 96 h post transfection and untrasfected Sf9 cells (10^6^ cells) by TRIZOL reagent.^20^ cDNA synthesis was performed with oligo (dT) and random hexamers primers according to the protocol provided by the Takara manufacturer. The synthesized cDNAs were amplified by PCR using specific primers (TG2-forward and TG2-reverse).

### Western blotting of TG2 recombinant

Following SDS–PAGE, the isolated proteins were transferred onto nitrocellulose membrane using a Bio-Rad wet blot system for 100 min at 100 V. The membranes were blocked with 2% BSA in PBS for overnight. After gently washing, the membranes were incubated with rabbit anti-TG2 antibody (1/2000, Abcam) for 1.5 h at RT. The detector layer was anti-rabbit IgG (1:10000, Santa Cruz Biotechnology). Finally, visualization was performed with DAB (Sigma).^21,22^

### Activity assay

The determination of TG2 activity was based on the incorporation of dansylcadaverin (CAD) (Sigma) as acyl acceptor (amine donor) into N,N dimethyl casein (Sigma) as acyl donor (amine acceptor) by fluorometric assay. Assay buffer containing 100 mM Tris (pH 8), 10 mM CaCl_2_, 0.2 mg/ml N,N dimethyl casein, 0.5 mM dansycadaverin and 10 mM DTT. EDTA (100 mM) was added to chelate Ca^2+^ ions. Recombinant human transglutaminase 2/TGM2 (1 μg) used for comparison of activities and as a positive control. Semipurified supernatant of 96 h post transfected and untransfected cell lysate were concentrated, and were added at 500 µg concentration. The reaction mixtures were incubated for 0, 30, 60, 120, and 180 min at 37 °C. The relative fluorescence of the reaction mixtures were measured in triplicate to achieve standard deviation using Bio-tek instrument (Synergy 4) at λ_exc_ 280 nm, λ_em_ 538 nm.^23^

To ensure that we detect TG2 activity, we performed the fluorometric with much longer incubation time for 16 h at 37 °C exactly as the aforementioned procedure except changing the EDTA concentration to 25 mM. After 16 h incubation, the reactions were stopped by adding 0.4M HCL and the samples were centrifuged to collect protein aggregates at 10000 rpm for 10 min. The pellets were washed by ethanol/acetone (1:1, v/v). Pellets air–dried and finally they were dissolved in 0.1M NaOH and 0.7M boric acid. The relative fluorescent of samples were measured at λ_exc_ 488 nm and λ_em_ 521 nm.^24^

For better confirming fluorescence activity assay, the reaction mixtures that were incubated at 180 min and 16 h in fluorometric assay, were subjected to SDS-PAGE (15%) at 100 V for 120 min (after adding Laemmli buffer and boiling for 5 min). The gels were photographed under UV light as previously performed.^24,25^ The gel was also stained by Coomassie brilliant blue R-250.

## Results

### Donor vector construction with a predesigned cassette (recombinant pFastBac HTA-TG2)

**Figure 1:**
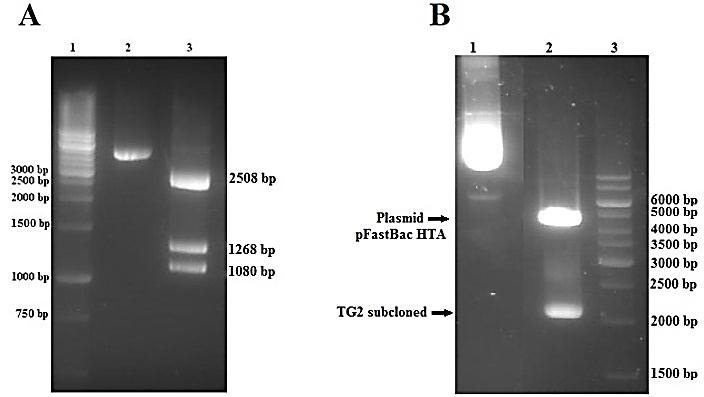
Verification of empty *pFastBac HTA* donor vector and TG2 subcloned *(pFastBac HTA-*TG2) by restriction enzymes. (A) Empty plasmid *pFastBac HTA* were cut with *Bgl I*. 1,1Kb DNA ladder; 2,undigested *pFastBac HTA* and 3,digested *pFastBac HTA*. (B) *pFastBac HTA-*TG2 digested with *EcoR I* and *Hind III*. 1,undigested *pFastBac HTA-*TG2, 2,digested *pFastBac HTA-*TG2 and 3,1Kb DNA ladder.

The digestion profile of plasmid *pFastBac HTA* using restriction enzyme *BglI* resulted in three fragments of 2508, 1268, 1080 base pairs, verified the donor vector (Figure 1A). For subcloning TG2 coding sequence^17^ into *pFastBac HTA* donor vector (*pFastBac HTA-*TG2*)*, recombinant cloning vector containing TG2 was digested by *EcoRI* and *Hind III* restriction enzymes (Figure 1B). After ligation and selection of correct constructs, DNA sequencing of recombinant *pFastBacHTA*-TG2 confirmed the existence of coding sequence in the correct frame of the vector, without any mutation or alteration in TG2 sequences.

### Analyzing Recombinant Bacmid DNA by PCR

**Figure 2:**
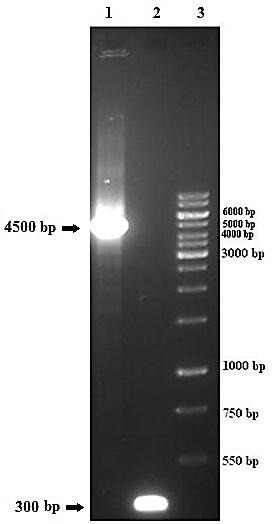
Transposition of TG2 into the bacmid supplied by DH10Bac was confirmed using pUC/M13 primers. 1,confirmed correct homologous recombination, 2,unrecombinant bacmid of DH10Bac as control negative and 3,1Kb DNA ladder.

PCR analysis was used to verify the presence of TG2 (gene of interest) in the recombinant bacmid using pUC/M13 forward and reverse primers. A PCR product near 4500 bp on the agarose gel verified the presence of TG2 coding sequence and correct homologous recombination in the bacmid (Figure 2).

### Expression analysis of the TG2 by RT-PCR, SDS-PAGE and Western blot

**Figure 3:**
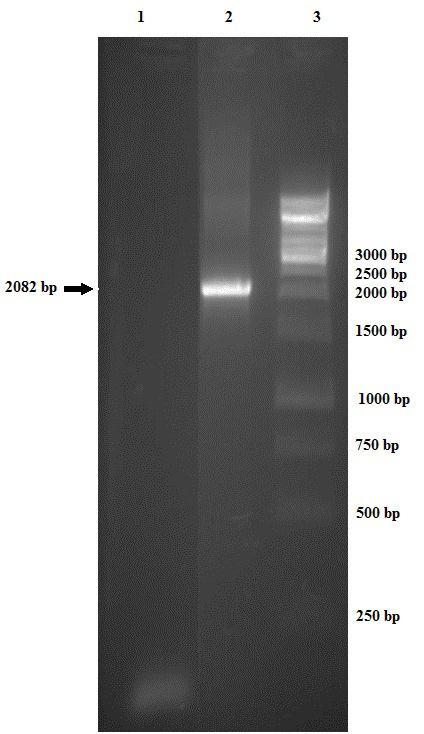
Detection of expressed TG2 at transcriptional level by RT-PCR. 1,no expression in untransfected Sf9 cells (control negative), 2,TG2 mRNA expression in 96 h post transfected Sf9 cells and 3,1Kb DNA ladder.

Contamination of the SF9 cell line with mycoplasma was investigated using universal specific primer.^26^ Moreover, cross-contamination and misidentification of this cell line were investigated using PCR and 13 human short tandem repeat (STR) primers (data not shown).^27^ After transfection of the bacmid, as explained in Materials and Methods, the expression of TG2 was detected at the transcriptional level. RT-PCR was used for mRNA expression after 96 h post infection using specific primers (TG2-forward and TG2-reverse). The results revealed that transcription of TG2 as a band near 2082 bp in the electrophoresis analysis (Figure 3).

**Figure 4:**
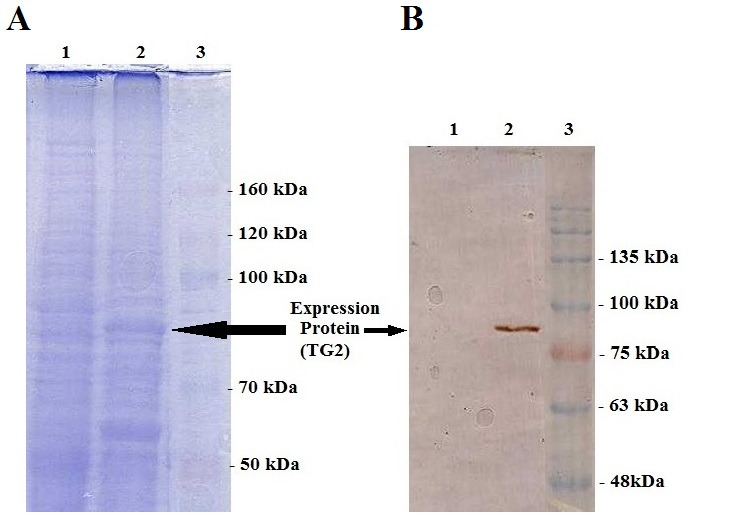
SDS-PAGE and Western blot of equivalent gel. (A) Coomassie brilliant blue-stained. (B) Western blot using monoclonal TG2 antibody. 1,total cell lysate of untransfected Sf9 as control negative; 2,total cell lysate of 96h post transfection Sf9 cells, and 3, protein molecular weight marker.

Expression of TG2 was also confirmed by SDS–PAGE and Western blot analysis after 96 h post infection. Figure 4-A shows the presence of a band measuring approximately 80 kDa in the infected cell with recombinant baculovirus, confirming the recombinant human TG2 presence. To further confirm TG2 expression, western blot analysis was performed. The same band reacted with the anti-human TG2, indicating that this band represents the recombinant human TG2 protein (Figure 4-B).

### Activity assay of recombinant TG2

The transamidation activity of TG2 was determined with high sensitivity by continuously following the relative rate of fluorescence. The increase in fluorescence resulting from covalent attachment of dansylcadaverine to N,N dimethyl casein was then determined.^24^ Dansylcadaverine has high affinity for TG enzymes; its incorporation into N,N dimethyl casein relates to an escalation in the quantum yield of dansyl group emission.^28^

**Figure 5:**
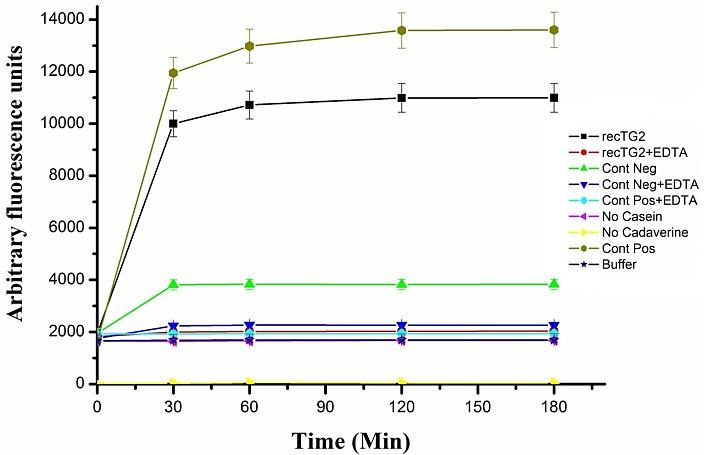
The activity of the recombinant TG2 was determined by fluorometric assay and measured in triplicate at Ex: 280 nm/ Em: 538nm. (■) transfected Sf9 cell lysate (rec TG2), (●) transfected Sf9 cell lysate (rec TG2) with 100 mM EDTA, (▲) untransfected (Cont Neg.) Sf9 cell lysat, (▼) untransfected (Cont Neg) Sf9 cell lysat with 100 mM EDTA, (♦) TGM2 (1 μg) with 100 mM EDTA, (◄) assay buffer without casein, (►) assay buffer without dansylcadaverine, (⬢)TGM2 (1 μg) as positive control and (∗) assay buffer as Blank.

After a time course (0-180 min) an increase in fluorescence can be observed. The fluorescence values in the transfected Sf9 cell lysate ( rec TG2) without EDTA in comparison with the corresponding controls, transfected Sf9 cell lysate (rec TG2) plus EDTA and untransfected Sf9 cell lysate (negative control) without EDTA, were greater than 2.5 to 5 folds, respectively (Figure 5). By adding EDTA (100 mM), the fluorescence intensity was greatly diminished, indicating that TG2 activity was inhibited by EDTA. Our data showed activity (~2 folds) in untransfeceted (Cont Neg) Sf9 cell without EDTA which could be probably due to the existence of endogenous transglutaminse of Sf9 insect cells^29^ (Figure 5).

**Figure 6:**
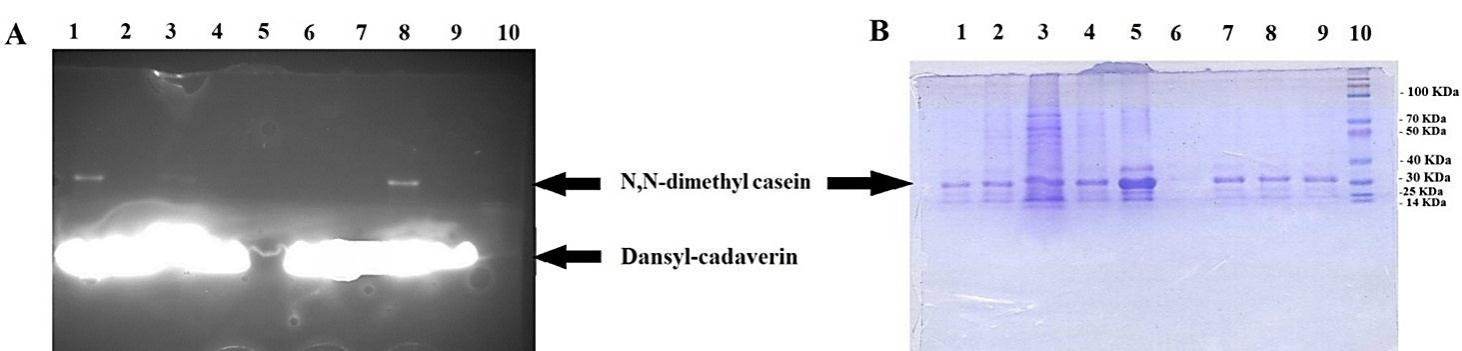
Fluorescence activity assay confirmed by SDS-PAGE (15%) incubation 180 min. A) Gel was photographed under UV light. B) the same gel stained by Coomassie brilliant blue. 1, transfected Sf9 cell lysate (rec TG2), 2, transfected Sf9 cell lysate (rec TG2) with 100 mM EDTA, 3, untransfected (Cont Neg) Sf9 cell lysate, 4, untransfected (Cont Neg) Sf9 cell lysate with 100 mM EDTA, 5, assay buffer without dansylcadaverine, 6, assay buffer without N,N dimethyl casein, 7, TGM2 with 100 mM EDTA, 8, TGM2 as positive control, 9, assay buffer as Blank and, 10, protein molecular weight marker.

In order to directly follow the activity of TG2, the activity was coupled with gel electrophoresis. Using this approach, the product of the enzyme can be directly detected by the gel analysis since the UV sensitive dansylcadaverine would be added to the substrate by the action of the enzyme. When the fluorescence assay was examined on the SDS-PAGE, the detection of UV sensitive substrate confirmed the actual cross linking reaction by the recombinant enzyme (Figure 6-A). After electrophoresis, the fluorescent band which migrated near 30 kDa, represented modified N,N dimethylcasein, linked covalently to densylcadaverine (Figure 6-B). The fluorescent band of the dansylcadaverine alone (335.46 Da) is depicted at the lower edge of the gel (Figure 6-A), exhibiting relatively high background fluorescence. The intensity of rec TG2 and control positive fluorescent bands were greatly reduced when EDTA was present in the reaction.

**Figure 7:**
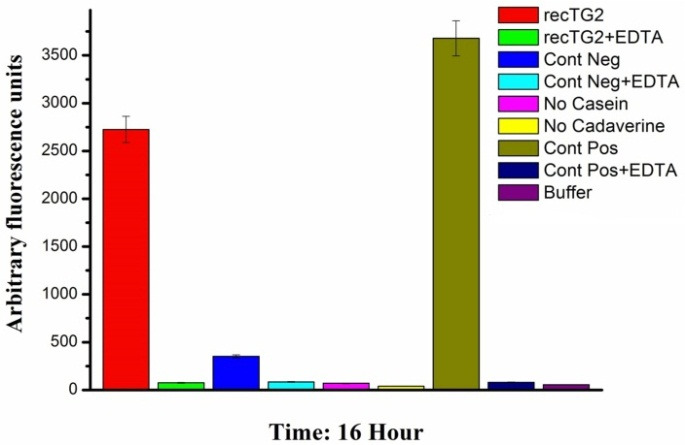
The activity of the recombinant TG2 was determined by fluorometric assay after 16 h. The relative fluorescence was measured in triplicate at Ex: 488 nm/ Em: 521nm. Columns from left to right: transfected Sf9 cell lysate (rec TG2), transfected Sf9 cell lysate (rec TG2) with 25 mM EDTA, untransfected (Cont Neg) Sf9 cell lysate, untransfected (Cont Neg) Sf9 cell lysate with 25 mM EDTA, assay buffer without casein, assay buffer without dansylcadaverine, TGM2 (1 μg) as positive control, TGM2 (1 μg) with 25 mM EDTA and assay buffer as Blank

**Figure 8:**
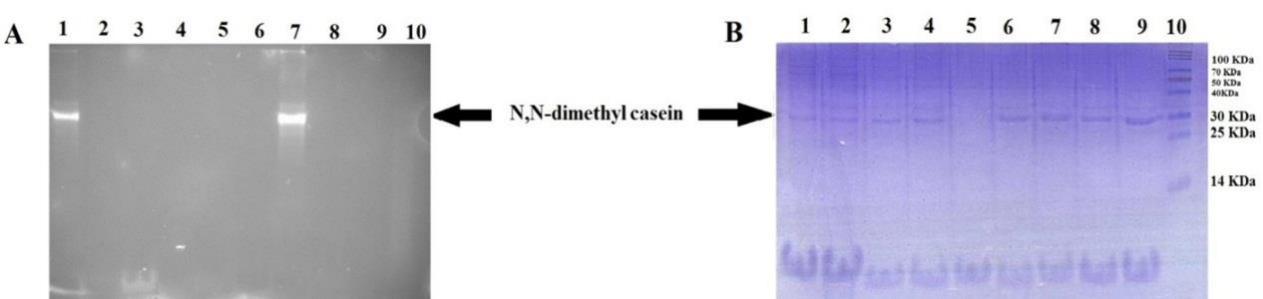
Fluorescence activity assay confirming by SDS-PAGE (15%) incubation 16 h. A) Gel photographed under UV light and B) the same gel stained by Coomassie brilliant blue. 1, transfected Sf9 cell lysate (rec TG2), 2, transfected Sf9 cell lysate (rec TG2) with 25 mM EDTA, 3, untransfected (Cont Neg) Sf9 cell lysate, 4, untransfected (Cont Neg) Sf9 cell lysate with 25 mM EDTA, 5, assay buffer without N,N dimethyl casein, 6, assay buffer without dansylcadaverine, 7, TGM2 with 25 mM EDTA, 8, TGM2 as positive control, 9, assay buffer as Blank, 10, protein molecular weight marker.

The fluorescence activity assay was also carried out in triplicate for 16 h at 37 °C. After 16 h, the reaction mixtures were precipitated for measuring its activity. The relatives fluorescence of transfected Sf9 cell lysate (rec TG2) in comparison with the corresponding controls, transfected Sf9 cell lysate (rec TG2) plus EDTA and untransfected (Control negative) Sf9 cell lysate without EDTA, were 250 and 5 folds, respectively (Figure 7). The huge difference between the incubation time indicates that the maximum activity for the enzyme is achieved in hours rather than in minutes. We also coupled the activity assay (after 16 h) with gel electrophoresis as shown in Figure 8. These results clearly confirmed the activity of the expressed recombinant TG2 in the presence of 10 mM Ca^2+^ as described previously.^17,23,24^

## Discussion

Tissue transglutaminase is the most abundantly expressed intracellular and extracellular enzyme of the transglutaminase family that catalyzes the Ca^2+^-dependent post-translational modification of proteins. TG2 is a unique and multifunctional enzyme, and because of its extensive interactions with numerous other gene products, the enzyme has a broad substrate specificity. Additionally, TG2 has shown non enzymatic (Ca^2+^-independent) activities which include cell growth,^11^ adhesion and differentiation,^30^ wound healing,^31^ and apoptosis.^5^ Multiple lines of evidence suggest an involvement of TG2 in disease pathology such as autoimmune diseases (celiac disease), cancer, and neurodegenerative diseases^12^ (Table 1). TG2 might be an attractive novel pharmacological target for treatment of neurodegenerative diseases in preventing toxic protein aggregation.^13^

Although many studies about the diverse aspects of tissue transglutaminase (TG2) specially its roles in pathobiology of diseases have been reported,^2,7,14^ there have not been much reports on the enzyme inhibitors. For this reason the expression of recombinant human TG2 enzyme as well as the assessment of its *in vitro* biological activity has been undertaken in the present study.

Expressions of human TG2 have already been reported in the bacterial expression systems. Shi* et al* expressed it in BL21(DE3) using the pET-30 Ek/LIC vector.^23^ Other investigators have also used *E. coli* expression system.^17,32^ Expressions in the aforementioned system have led to insoluble inclusion bodies leading to the lack of activity for the enzyme. There have been some attempts to decrease the TG2 formation of inclusion bodies and increase the yield of soluble active enzyme.^17,23^ Because the bacterial expression systems do not carry out post-translational modifications and lack special organelles as compared to mammalian systems, this discrepancy could be the main reason for obtaining nonfunctional human recombinant proteins such as TG2 recombinant proteins. Moreover, contamination of recombinant protein with endotoxin which is often seen with the microbial expression system restrict their applications for *in vivo* studies.^33^

One of the most common eukaryotic expression systems is the baculovirus expression vector system (BEVS). BEVS has high levels of heterologous gene expression compared to the other eukaryotic expression systems, particularly for intracellular proteins. Baculoviruses have a restricted host range which are limited to specific invertebrate species; therefore, these viruses are safer to work in comparison with most mammalian viruses. This expression system has also a number of potential benefits including correct folding and post-translational modification of the recombinant proteins.^16,33-35^ Among the various methods of BEVS, Bac-to-Bac technique is a rapid, efficient and widely used method.^16^ Our previous data showed Bac-to-Bac is more efficient for large scale production and has been widely used for mammalian recombinant proteins.^18,19^ Osman *et al* produced recombinant human tissue transglutaminase carried a C-terminal His tag in TriEx baculovirus expression system and did not assay its activity.^36^ For identifying the recombinants in TriEx baculovirus expression system (Novagen), plaque screening and often additional rounds are needed to guarantee that the recombinant viral preparation are not contaminated with wild-type virus. By using site specific transposition in Bac-to-Bac technique, one can overcome the requirement to isolate plaques and the efficiency of recombination in this system is nearly 100%.^33,37^ Because of lengthy and laborious process for generating recombinant in TriEx expression system, we considered the expression of this enzyme (TG2) using Bac-to-Bac technique. To best of our knowledge, this is the first report of expression the recombinant human TG2 by Bac-to-Bac baculovirus expression system.

The cDNA of human tissue transglutaminase^17^ was used for baculovirus Bac-to-Bac technique. Sf9 cells were negative for mycoplasma and their STR profiles confirmed no cross-contamination and misidentification of this cell line. The donor pFastBac HTA vector would add extra 6xHis-Tag sequence at N-terminal of recombinant protein,^19^ enabling us to use Nickel -affinity column chromatography for purifying recombinant protein. TG2 expression process was confirmed by RT-PCR (Figure 3), SDS-PAGE (Figure 4-A) and western blotting (Figure 4-B). Functional analyses revealed the enzyme had biological activity (Figure 5 and 7). For directly visualizing crosslinking activity of TG2 enzyme, SDS-PAGE analyses were performed to reaffirm the functionality of the expressed enzyme (Figure 6 and 8). In all activity assays experiments, EDTA (100 mM or 25 mM), as a chelating agent for calcium ions, to deactivate the enzymatic activity of TG2 (Figure 5,6,7 and 8).

## Conclusion

TG2 was expressed efficiently by Bac-to-Bac expression system. Our data show that the produced recombinant protein (TG2) in this expression system possessed a considerable biological activity. The procedure of expression in this study is expected to be applicable for large-scale production of recombinant TG2 in baculovirus system. The further goals of this project will be purifying of TG2 and characterize its role in a number of biological networks. The expressed protein also could be used for diagnostic diseases, in production of antibody against TG2, and can be exploited searching for novel inhibitors of the enzyme as potential therapeutic approach in future.

## Acknowledgments

We wish to thank to Dr. Mohammad Ali Shokrgozar of National Cell Bank of Iran (NCBI), Pasteur Institute of Iran his continuous support. This study were funded by Golestan University of Medical Sciences, (Award No: 920501049) and a grant from Sharif University of Technology (Grant No: 6930614).

## Ethical Issues

Not applicable.

## Conflict of Interest

The authors report no conflicts of interest.
